# The association between nutrient patterns and hospital stay duration and symptoms in COVID-19 in Iranian patients: cross-sectional study

**DOI:** 10.3389/fnut.2025.1542449

**Published:** 2025-03-03

**Authors:** Atieh Mirzababaei, Farideh Shiraseb, Azam Mohamadi, Mahya Mehri Hajmir, Sara Ebrahimi, Zeinab Zarrinvafa, Elham Kazemian, Amir Mehrvar, Khadijeh Mirzaei

**Affiliations:** ^1^Chronic Diseases Research Center, Endocrinology and Metabolism Population Sciences Institute, Tehran University of Medical Sciences, Tehran, Iran; ^2^Department of Community Nutrition, School of Nutritional Sciences and Dietetics, Tehran University of Medical Sciences, Tehran, Iran; ^3^Imam Khomeini Hospital Complex, Tehran University of Medical Sciences, Tehran, Iran; ^4^Department of Exercise and Nutrition Sciences, Milken Institute School of Public Health, The George Washington University, Washington, DC, United States; ^5^Institute for Physical Activity and Nutrition, School of Exercise and Nutrition Sciences, Deakin University, Geelong, VIC, Australia; ^6^Department of Nutrition, Science and Research Branch, Islamic Azad University, Tehran, Iran; ^7^Department of Medicine, Samuel Oschin Comprehensive Cancer Institute, Cedars-Sinai Medical Center, Los Angeles, CA, United States; ^8^Department of Orthopedics, Taleghani Hospital Research Development Committee, Shahid Beheshti University of Medical Sciences, Tehran, Iran

**Keywords:** nutrient patterns, COVID-19, obesity, signs and symptoms, length of hospital stay

## Abstract

**Background:**

An excessively reactive immune system results in the cytokine storm COVID-19. A healthy diet is essential to maintain the balance between the immune system and inflammatory and oxidative stress. Associations between single foods and nutrients and COVID-19 have been examined. However, no prior study has examined associations between nutrient patterns and COVID-19. This study assessed the link between nutrient patterns and the COVID-19 severity and length of hospital stay in Iranian adults.

**Methods:**

This cross-sectional study included 107 Iranian adults aged 20–60 years, who were admitted to Amir Alam Hospital in Tehran, Iran, due to COVID-19. Data on their symptoms were collected through a demographic questionnaire and verified against their hospital records. Three non-consecutive 24-h dietary recalls were used to collect participants’ food and beverage intake. Principal component analysis (PCA) was used to derive nutrient patterns.

**Result:**

A total of 95 Covid patients with a mean age of 46.2 years were included. Four major dietary patterns were identified using the Scree Plot chart, including high carbohydrate and high minerals pattern; high protein and high vitamins pattern; high fat pattern; and poor nutrient pattern. Adherence to the poor nutrient patterns was associated with a higher number of hospitalization days and lower appetite (*p* < 0.05). The poor dietary patterns were associated with an increased likelihood of headache, fever, and respiratory distress syndrome (RDS). Also, headaches were more common with adherence to the high-fat pattern (*p* < 0.05).

**Conclusion:**

The findings of this study show that a poor nutrient pattern is related to longer hospital stays and reduced appetite. It also connected to an increased likelihood of symptoms including headaches, fever, and respiratory distress syndrome. A strong association was found between respiratory distress syndrome, headaches, and a high-fat diet was found. Further studies with prospective designs are needed to better understand and validate these findings.

## 1 Introduction

Coronavirus Disease (COVID-19) originated from a strain known as severe acute respiratory syndrome coronavirus 2 (SARS-CoV-2). COVID-19 was first detected in Wuhan, China, and spread to 190 countries (1, 2). Iran was one of the country’s most severely affected by COVID-19, with approximately 6.7 million COVID-19 cases, placing it twelfth globally. Officially confirmed deaths from COVID-19 in Iran reached 133′000 in 2021 (3–5). While most individuals infected with COVID-19 undergo mild to moderate respiratory symptoms, some people may experience severe symptoms (6). The most common COVID-19 symptoms are fever, cough, tiredness, and loss of taste or smell (6). The COVID-19 pandemic had a high burden globally, including death, severe respiratory diseases, hospital stays, restrictive policies such as lockdowns, and personal movement limitations, which resulted in social and economic problems and global crises (7, 8).

The cytokine storm in individuals infected with COVID-19, stems from a weakened or excessively reactive immune system (9, 10). Various factors such as genetics, physical activity, stress, vaccination status, and diet could influence the immune system. The COVID-19 pandemic has highlighted the importance of nutrition in modulating the immune response and influencing disease severity. Emerging evidence suggests that various nutrients play a pivotal role in immune function, which can directly affect the course of the disease. A diet rich in vitamins, minerals, polyphenols, and antioxidants has been identified as essential for maintaining immune health and potentially mitigating the severity of COVID-19 (11, 12). The World Health Organization (WHO) considers a healthy diet an important factor during COVID-19 (7, 13). A healthy diet is essential to maintain the balance between the immune system and inflammatory and oxidative stress processes (14, 15). Food groups with abundant phytochemicals, polyphenols, and fiber, are recommended to foster the growth of beneficial bacteria, which alleviates diarrhea, a common symptom of COVID-19 (16, 17). Furthermore, micronutrients, including vitamins A, folic acid, vitamin B6, vitamin B12, vitamin D, vitamin C, selenium, zinc, and iron have been consistently reported for their role in proper immune function (18). These micronutrient deficiencies could reduce the resistance to infection and recovery from it (18, 19).

Associations between single foods such as fruits and vegetables, flax seeds, basil, and COVID-19 have been previously examined (20, 21). While understanding the role of a single food is important, a diet consists of various foods and nutrients consumed together. As a result, it is worth assessing a whole diet in relation to health outcomes (22, 23). Dietary pattern methodology enables researchers to achieve a holistic understanding of diets. Some studies have explored the relationship between pre-disease dietary patterns and the severity of COVID-19 symptoms (24). A study on 592,571 adults from the US and UK reported an inverse link between the healthful Plant-Based Diet Index (HPDI) and COVID-19 risk and severity (25). Another study applied empirical dietary patterns on 1,106 Iranian adults and found that participants with higher adherence to Western dietary patterns were more likely to contract COVID-19 (26). These studies suggest that dietary habits prior to infection can play a significant role in the outcome of COVID-19. For instance, a study by Shakeri et al. (2021) indicated that individuals adhering to a Mediterranean diet, which is rich in fruits, vegetables, and healthy fats, had a lower risk of severe COVID-19 symptoms and hospitalizations (27). Similarly, another study found that a plant-based diet, characterized by high consumption of fiber, vitamins, and antioxidants, was inversely associated with COVID-19 severity (28). These findings underscore the potential importance of pre-disease nutrition in influencing the clinical course of COVID-19, suggesting that habitual dietary choices may modulate immune response and inflammation, both of which are critical in the development and progression of the disease.

Furthermore, several studies assessed associations between single nutrients such as vitamin D, vitamin A, vitamin C, zinc and COVID-19 severity and length of hospital stay (29–33). A study on 295 Iranian adults diagnosed with COVID-19 investigated associations between the dietary antioxidant quality score (DAQS) and the severity of COVID-19. DAQS is comprised of limited nutrients including vitamin E, vitamin A, vitamin C, selenium, and zinc. This study found that a stronger adherence to DAQS was linked to a significant decrease in the severity of COVID-19 infection (34). Considering the important role of nutrients in COVID-19 and the interaction between nutrients that make up a diet, investigating the overall nutrient pattern associated with COVID-19 may be more beneficial (35–37). However, no prior study has comprehensively examined the associations between nutrient patterns and COVID-19 outcomes. This study, for the first time, re-examines the relationship between nutrient patterns and COVID-19 severity, as well as the length of hospital stay, using a cross-sectional design and analyzing dietary patterns and hospitalization data from 107 Iranian adults. By incorporating a more holistic approach to nutrient interactions, this study aims to provide new insights specific to the Iranian population.

## 2 Methods

### 2.1 Study design and population

This cross-sectional study recruited 107 Iranian adults with COVID-19 who were referred to Amir Alam Hospital in Tehran, Iran, in 2020 using a random sampling method. The inclusion criteria were age between 20–60 years old, COVID-19 symptoms including fever, chills, sore throat, tiredness, sneezing, hard breathing, chest pain and fatigue and other common symptoms. Data on symptoms were collected through a demographic questionnaire and hospital records, with laboratory-confirmed COVID-19 infection using reverse transcription polymerase chain reaction (RT-PCR) testing of nasopharyngeal and oropharyngeal samples, biochemical evaluation that included CRP and D-dimer. WHO interim guidance which is Standard confirmation of acute SARS-CoV-2 infections was used for COVID-19 diagnosis. The exclusion criteria were liver and kidney diseases, any rare viruses including HIV, and chemotherapy within the past month. A well-trained nutritionist conducted all interviews and completed the questionnaires.

### 2.2 Sociodemographic characteristics, anthropometric indices and physical activity

A questionnaire was used to collect data on age, sex, marital status, education, and job status. A Seca digital scale (Germany) with a precision of 0.1 kg was used to assess participants’ body weight. Height was measured using a Seca 206 stadiometer (Germany) with a precision of 0.1 cm. The International Physical Activity Questionnaire (IPAQ) was used to assess physical activity (PA) of participants (38).

### 2.3 Dietary intake assessment and nutrient pattern

Three non-consecutive 24-h dietary recalls were used to collect participants’ food and beverage intake over two-week days and one weekend. We ensured that the 24-h dietary recalls were not based on hospital meals. Instead, these recalls were obtained by interviewing the patient’s companion or family member, focusing specifically on the participant’s dietary intake prior to hospitalization. This method was used to reflect habitual dietary patterns before the acute event that led to hospitalization. The FFQ was not feasible during the acute phase of the pandemic due to time constraints and limited participant engagement, while the 24-h recall allowed for reliable, bias-minimized. The nutrients and energy intake were measured using NUTRITIONIST-IV (version 7.0; N Squared Computing, Salem, OR, USA) which is based on the USDA food composition database. Principal component analysis (PCA) with varimax rotation was used to derive nutrient patterns. A total of 37 nutrients were selected to derive nutrient patterns. The number of nutrient patterns was identified if eigenvalues>1.5 using the scree plots and based on previous Iranian surveys (33–35). Patterns were described on the nutrients that loaded most positively or negatively for each specific pattern.

After data analysis using the PCA method and based on data related to food intake, the suitability of the data collected for PCA analysis was examined using a review of the Anti-Image table, the results of the Kaiser-Meyer-Olkin (KMO) measure of sampling adequacy test, and Bartlett’s Test of Sphericity. KMO index was 0.89. Nutrient patterns were determined by selecting factors with a load factor greater and lower than 0.2, along with Eigenvalues greater than 1.5.

### 2.4 Discharge criteria for COVID-19 patients

The discharge criteria for COVID-19 patients include being medically stable and ready for discharge; improvement of respiratory symptoms; and being fever-free for at least 24 h without the use of antipyretics (e.g., Tylenol, NSAIDs, etc.). Furthermore, appropriate housing, transportation, and wrap-around services (food, medication, basic needs such as clothing and toiletries, and other necessities) must be in place to maintain isolation/transmission (Table 1).

**Table 1:** Factor loadings for four nutrient patterns identified by principal component analysis.

Nutrients (*n* = 37)	High carbohydrate, high minerals pattern	High protein, high vitamins pattern	High fat pattern	Poor in nutrients pattern
Carbohydrate	0.466			
Dietary Fiber	0.587			
Zinc	0.631			
Copper	0.718			
Iron	0.649			
Manganese	0.843			
Magnesium	0.785			
Selenium	0.284			
Thiamin B1	0.729			
Niacin B3	0.502			
Folate	0.721			
Cobalamin B12		0.816		
Riboflavin B2		0.809		
Protein		0.805		
Phosphorus		0.704		
Calcium		0.672		
Pantothenic Acid		0.569		
Saturate Fat		0.567		
Potassium		0.555		
Cholesterol		0.553		
Sodium		0.527		
Pyridoxine B6		0.428		
Vitamin A		0.316		
Beta Carotene		0.278		
Fat			0.862	
Poly unsaturated fat			0.850	
Mono unsaturated fat			0.784	
Tocopherol			0.777	
Linoleic acid			0.538	
Vitamin E			0.411	
Sugar				0.667
Molybdenum				0.620
Vitamin K				0.604
Biotin				0.584
Chromium				0.500
Vitamin C				0.469
Linolenic acid				0.251

Factor-loadings for major dietary patterns. Factor loadings below <0.20 were excluded for simplicity. Eigenvalues>1.5, Kaiser-Meyer-Olkin (KMO) index: 0.89.

### 2.5 Data analysis

The data were analyzed using SPSS software version 21. *p*-value <0.05 was considered statistically significant. *p*-values between 0.05 and 0.07 were considered marginally significant. The normality of continuous variables was assessed using the Kolmogorov–Smirnov test (*p* > 0.05). Mean and standard deviation (SD) were reported for continuous variables while numbers and percentages were reported for categorical variables. One-way analysis of variance (ANOVA) and chi-square were used to compare continuous and categorical variables, respectively. Analysis of covariance (ANCOVA) was used for the analysis adjusted for confounders. Nutrient patterns were categorized into lower and higher adherence using a median. Linear Regression analysis was used to assess associations between Hospitalization days containing DH (day) and Recovery time (day), signs containing C reactive-protein (CRP) (mg/L) and D-dimer (ng/ml), symptoms containing smell and taste and appetite and lethargy. A binary logistic regression analysis was used to assess associations between hospitalization days, recovery time, CRP, D-dimer, smell, taste, appetite, and lethargy. Model 1 was adjusted for age, sex, education level, BMI and physical activity. Model 2 was adjusted for age, sex, education level, BMI, physical activity, comorbidity, use of medication, and supplementation intake. In binary logistic regression chest pain (No), headache (No), nose (No), vomiting (No), nausea (No), diarrhea (No), sore throat (No), stomach pain (No), joint pain (No), confusion (No), contusion (No), chills (NO), RDS (No) considered as reference group. For statistical analysis, confidence intervals (CIs) were calculated for all reported estimates to provide a range of plausible values for the population. CIs allow for a more comprehensive interpretation of results, indicating the uncertainty surrounding the estimated values. Additionally, *p*-values were used to assess the statistical significance of associations. For marginally significant *p*-values (close to 0.05), the potential implications of these findings were carefully considered and discussed. This approach helps to avoid overstating the significance of borderline results and acknowledges the need for further research to confirm these associations.

## 3 Results

### 3.1 Study population

The study revealed that the mean (± SD) age, weight, and BMI were 46.21 (1.10) years, 86.75 (1.31) kg, and 29.36 (0.35) kg/m^2^, respectively. Most participants were married (85%) and men (66%). The maximum duration of hospitalization was 17 days, with a maximum recovery time of 3 weeks (Table 2).

**Table 2:** Characteristics of participants across nutrient patterns groups (*n* = 107).

Variable	High carbohydrate, high minerals pattern				High protein, high vitamins pattern				High fat pattern				Poor in nutrients pattern			
	<0.048	≥0.048	*p-value*	p-value^*^	<−0.17	≥ −0.17	*p-value*	p-value^*^	<0.13	≥0.13	*p-value*	p-value^*^	<−0.08	≥−0.08	*p-value*	p-value^*^
Demographic variables																
Age (year)	46.33 ± 11.08	46.19 ± 11.51	0.95	0.77	47.38 ± 11.20	45.16 ± 11.28	0.33	0.20	44.70 ± 10.93	47.85 ± 11.44	0.17	0.30	46.54 ± 11.06	45.54 ± 11.53	0.80	0.39
Anthropometric indices																
Weight (kg)	86.41 ± 14.93	87.41 ± 12.29	0.72	0.71	88.42 ± 14.10	85.42 ± 13.12	0.28	0.14	88.79 ± 13.70	84.98 ± 13.41	0.17	0.46	86.92 ± 14.50	86.92 ± 14.50	0.99	0.54
Height (cm)	171.27 ± 8.23	171.05 ± 7.32	0.89	0.11	171.53 ± 8.49	170.80 ± 7.02	0.65	**0.01**	173.31 ± 6.73	168.97 ± 8.17	**<0.01**	0.09	169.34 ± 7.79	173.02 ± 7.33	**0.02**	0.69
BMI (kg/m^2^)	29.33 ± 3.73	29.85 ± 3.61	0.49	0.20	27.64 ± 1.79	31.25 ± 4.21	0.25	0.62	29.46 ± 3.49	29.71 ± 3.85	0.73	0.95	30.20 ± 3.87	28.95 ± 3.36	0.09	0.46
PA (met/s)	151.6 ± 356.9	358.6 ± 1.75.5	0.20	0.15	314.8 ± 1067.9	194.4 ± 397.3	0.46	0.16	201.5 ± 723.9	307.6 ± 876.2	0.52	0.43	254.5 ± 787.4	253.5 ± 822.4	0.99	0.96
Categorical variables *N* (%)																
Sex																
Male	31 (48.4)	33 (51.6)	0.66	0.67	28 (43.8)	36 (56.3)	0.12	0.15	39 (60.9)	25 (39.1)	**<0.01**	**0.02**	27 (42.2)	37 (57.8)	**0.02**	**0.01**
Female	17 (54.8)	14 (45.2)			19 (61.3)	12 (38.7)			9 (29.0)	22 (71.0)			21 (67.7)	10 (32.3)		
Marriage status																
Married	46 (54.1)	39 (45.9)	**0.05**	0.17	42 (49.4)	43 (50.6)	1.00	0.60	43 (50.6)	42 (49.4)	1.00	0.94	43 (50.6)	42 (49.4)	1.00	0.76
Single	2 (22.2)	7 (77.8)			4 (44.4)	5 (55.6)			5 (55.6)	4 (44.4)			4 (44.4)	5 (55.6)		
Occupation categories																
Self-employment	19 (55.9)	15 (44.1)	0.95	0.36	16 (47.1)	18 (52.9)	0.78	0.37	22 (64.7)	12 (35.3)	0.07	0.39	16 (47.1)	18 (52.9)	0.08	0.15
Office work	11 (47.8)	12 (52.2)			10 (43.5)	13 (56.5)			12 (52.2)	11 (47.8)			8 (34.8)	15 (65.2)		
Educational work	2 (33.3)	4 (66.7)			2 (33.3)	4 (66.7)			1 (16.7)	5 (83.3)			3 (50)	3 (50)		
Unemployed	4 (50)	4 (50)			4 (50)	4 (50)			5 (62.5)	3 (37.5)			5 (62.5)	3 (37.5)		
Other^1^	11 (50)	11 (50)			14 (63.6)	8 (36.4)			8 (36.4)	14 (63.6)			16 (72.7)	6 (27.3)		
Education																
Undergraduate^2^	21 (46.7)	24 (53.3)	0.72	0.35	24 (53.3)	21 (46.7)	0.20	0.96	26 (57.8)	19 (42.2)	0.50	0.25	24 (53.3)	21 (46.7)	0.53	0.10
Medication use																
Yes	23 (53.5)	20 (46.5)	0.68	0.46	23 (53.5)	20 (46.5)	0.53	0.24	20 (46.5)	23 (53.5)	0.53	0.55	20 (46.5)	23 (53.5)	0.53	0.68
No	25 (48.1)	27 (51.9)			24 (46.2)	28 (53.8)			28 (53.8)	24 (46.2)			28 (53.8)	24 (462)		
Supplements intake																
Yes	14 (58.3)	10 (41.7)	0.25	0.25	12 (50)	12 (50)	0.57	0.74	11 (45.8)	13 (54.2)	0.38	0.77	13 (54.2)	11 (45.8)	0.43	0.77
No	34 (47.9)	37 (52.1)			35 (49.3)	36 (50.7)			37 (52.1)	34 (47.9)			35 (49.3)	36 (50.7)		
History of diseases																
Yes	24 (53.3)	21 (46.7)	0.39	0.41	21 (46.7)	24 (53.3)	0.83	0.06	22 (48.9)	23 (51.1)	0.83	0.43	22 (48.9)	23 (51.1)	1.00	0.91
No	19 (43.2)	25 (56.8)			22 (50)	22 (50)			23 (52.3)	21 (47.7)			22 (50)	22 (50)		

SD, standard deviation; BMI, Body mass index; PA, physical activity. Values are mean ± standard deviation (SD) and; numbers and percentage for categorical variables. One-way ANOVA was used for continuous variables. ^*^Adjusted for energy intake, age and physical activity. *p*-value < 0.05 significant and statistically significant results are provided in bold values. In addition, *p*-values between 0.05 to 0.07 were considered marginally significant. ^1^Other: any job other than the ones that have already been mentioned. ^2^Graduated and post graduated were considered as reference.

**Figure 1:**
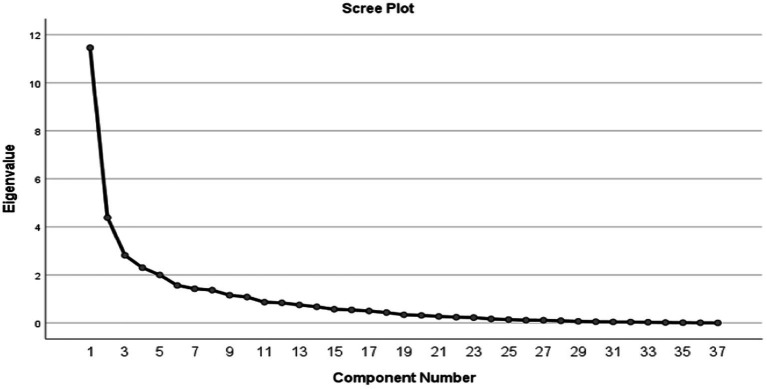
Scree plot chart for extracting major dietary patterns from principal component analysis.

According to the Scree Plot chart, four major dietary patterns were identified, including high carbohydrate and high minerals pattern; high protein and high vitamins pattern; high fat pattern; and poor nutrient pattern (Figure 1).

### 3.2 Demographic characteristics across nutrient patterns categories

Demographic characteristics across nutrient patterns are illustrated in Table 2. There was a significant mean difference in height between higher and lower adherence to high protein and high vitamins pattern after adjusting for age, BMI, and energy intake (*p* = 0.01). Furthermore, a significant mean difference in height was found between lower and higher adherence to high fat pattern (*p* < 0.01) and poor nutrient pattern (*p* = 0.02) in the crude model. However, after controlling for confounders, this significant association disappeared (*p* > 0.05). In the crude model and after adjustment for confounding variables, a significant difference was found in lower and higher adherence to the high fat and poor nutrient pattern between females and males (*p* < 0.05). The statistical analysis revealed significant associations between the identified nutrient patterns and COVID-19 severity. Confidence intervals (CIs) for all estimates were provided to offer a range of plausible values. For some associations, *p*-values were marginally significant (close to 0.05). These findings should be interpreted with caution, as further studies with larger sample sizes are needed to confirm the associations observed. The inclusion of CIs allows for a more nuanced understanding of the results and provides insight into the precision of the estimates.

### 3.3 Associations between nutrient patterns and symptoms and duration of hospitalization from COVID-19

The association between nutrient patterns and symptoms and duration of hospitalization from COVID-19 is shown in Table 3. A higher adherence to poor nutrient patterns was negatively associated with appetite in the crude model (*β*: −1.56; 95% CI: −2.74, −0.38), and in the adjusted model 1 (β: −1.55; 95% CI: −2.83, −0.27) and Model 2 (*β*: −1.65; 95% CI: −2.91, −0.39) (P:0.01). There was a marginally significant positive association between adherence to poor nutrient patterns and hospitalization days in the crude model (β: 1.01; 95% CI: −0.10,2.13) and in model 2 (β:1.12; 95% CI: −0.13,2.38) (*p* < 0.05). Furthermore, in model 2, higher adherence to high carbohydrate and high minerals nutrient patterns was negatively associated with recovery time (β: −1.31; 95% CI: −3.28, 0.64) (*p* < 0.05).

**Table 3:** Associations between nutrient patterns and signs and symptoms and duration of hospitalization of COVID-19 (*n* = 107).

	High carbohydrate, high minerals pattern <−0.04	*p-value*	High protein, high vitamins pattern <−0.17	*p-value*	High fat pattern <0.13	*p-value*	Poor in nutrients pattern <−0.08	*p-value*
*β* (95%CI)								
Variables								
Hospitalization days DH (day)								
Crude	−0.03 (−1.14,1.13)	0.96	−0.003 (−1.1,1.13)	0.99	0.97 (−0.15,2.09)	0.09	1.01 (−0.10,2.13)	0.07
Model 1	0.21 (−1.00,1.43)	0.73	0.07 (−1.14,1.29)	0.90	0.88 (−0.33,2.10)	0.15	0.99 (−0.24,2.23)	0.11
Model 2	0.32 (−0.94,1.59)	0.61	0.25 (−1.00,1.50)	0.69	0.83 (−0.38,2.05)	0.17	1.12 (−0.13,2.38)	**0.05**
Recovery time (day)								
Crude	0.18 (−1.78,2.15)	0.85	0.18 (−1.78,2.15)	0.85	0.19 (−1.77,2.16)	**0.03**	−0.01 (−1.98,1.95)	0.98
Model 1	−0.83 (−2.83,1.16)	0.41	−0.40 (−2.40,1.59)	0.69	0.63 (−1.39,2.65)	0.54	−0.40 (−2.46,1.64)	0.69
Model 2	−1.31 (−3.28,0.64)	**0.05**	0.18 (−1.76,2.14)	0.85	0.63 (−1.28,2.54)	0.51	0.05 (−1.94,2.05)	0.95
Sign								
CRP (mg/L)								
Crude	−16.33 (−180.6,147.9)	0.84	−16.33 (−180.6,147.9)	0.84	−30.7 (−194.9,133.4)	0.71	20.45 (−143.7,184.8)	0.80
Model 1	−18.45 (−191.0,154.1)	0.83	14.20 (−23.64,52.04)	0.45	−32.92 (−70.61,4.76)	0.08	−21.23 (−59.00,16.53)	0.27
Model 2	−37.55 (−206.0,130.95)	0.66	−83.78 (−248,81.37)	0.32	−80.47 (−242.4,81.53)	0.33	−29.34 (−199.0140.3)	0.75
D-dimer (ng/ml)								
Crude	113 (−82.56,309.0)	0.25	113.22 (−82.5,309.0)	0.25	53.64 (−143.1,250.4)	0.59	11.02 (−186.0,208.1)	0.91
Model 1	123.96 (−75.81,323.7)	0.22	1.90 (−5.64,9.45)	0.62	−1.10 (−9.16,6.96)	0.78	−5.84 (−1.78,2.09)	0.14
Model 2	140.43 (−67.75,348.4)	0.18	17.56 (−189.2,224.3)	0.86	2.17 (−200.6,204.9)	0.98	−71.87 (−282.7,139.0)	0.50
Symptoms								
Smell								
Crude	0.25 (−1.24,1.76)	0.73	0.25 (−1.24,1.76)	0.73	−0.17 (−1.68,1.33)	0.82	0.03 (−0.47,1.54)	0.96
Model 1	−0.39 (−2.01,1.21)	0.62	0.01 (−1.60,1.62)	0.99	−0.12 (−1.75,1.50)	0.88	−0.61 (−2.26,1.03)	0.46
Model 2	−0.16 (−1.81,1.49)	0.85	0.12 (−1.50,1.76)	0.87	0.81 (−1.78,1.41)	0.81	−0.58 (−2.24,1.08)	0.49
Taste								
Crude	0.94 (−0.53,2.41)	0.21	0.94 (−0.53,2.41)	0.21	1.24 (−0.21,2.71)	0.09	−1.02 (−2.49,0.44)	0.17
Model 1	−0.09 (−1.68,1.50)	0.91	0.83 (−0.74,2.41)	0.30	0.93 (−0.66,2.53)	0.25	−0.46 (−2.09,1.17)	0.58
Model 2	−0.14 (−1.45,1.74)	0.71	1.21 (−0.43,2.67)	0.15	0.79 (−0.74,2.35)	0.31	−0.45 (−2.05,1.15)	0.58
Appetite								
Crude	0.26 (−0.96,1.48)	0.65	0.26 (−0.96,1.48)	0.65	−0.64 (−1.85,0.57)	0.30	−1.56 (−2.74, −0.38)	**0.01**
Model 1	0.54 (−0.73,1.82)	0.40	−0.06 (−1.35,1.21)	0.91	−0.26 (−1.56,1.03)	0.69	−1.55 (−2.83, −0.27)	**0.01**
Model 2	0.95 (−0.32,2.24)	0.14	0.29 (−0.98,1.56)	0.65	−0.39 (−1.64,0.85)	0.53	−1.65 (−2.91, −0.39)	**0.01**
Lethargy								
Crude	0.28 (−0.83,1.41)	0.61	0.28 (−0.83,1.41)	0.61	0.68 (−0.44,1.80)	0.23	0.17 (−0.95,1.30)	0.76
Model 1	0.61 (−0.58,1.81)	0.31	0.39 (−0.80,1.58)	0.52	0.40 (−0.80,1.61)	0.51	0.25 (−0.981.48)	0.69
Model 2	0.64 (−0.58,1.88)	0.30	0.56 (−0.64,1.78)	0.35	0.37 (−0.81,1.57)	0.53	0.13 (−1.11,1.38)	0.83

DH, Duration of hospitalization; RDS, Respiratory distress syndrome; CRP, C-reactive protein. Model 1 was adjusted for age, sex, education level, BMI and physical activity. Model 2 was adjusted age, sex, education level, BMI, physical activity, comorbidity, use of medication, and supplementation use. Nutrient patterns were reported as median. High carbohydrate, high minerals ≥ −0.08, high protein, high vitamins ≥ 0.013, high fat ≥ −0.017 and Poor in nutrients ≥ −0.04 are reference categories. Data were obtained from the linear regression. Data are presented as β and 95% confidence interval. Significant *p* value < 0.05 are bolded. In addition, *p*-values between 0.05 and 0.07 were considered marginally significant. Linear Regression analysis was used to assess associations between Hospitalization days containing DH (day) and Recovery time (day), signs containing CRP (mg/L) and D-dimer (ng/ml), Symptoms contains Smell and taste and Appetite and Lethargy.

### 3.4 Associations between nutrient patterns and COVID-19 symptoms

The association between nutrient patterns and COVID-19 symptoms is presented in Table 4. In the crude model, individuals with higher adherence to poor nutrient patterns were significantly associated with higher odds of chest pain (OR: 0.88; 95% CI: 0.01,1.72) and in model 2 (OR:2.26; 95% CI: 0.88,5.79). Participants with higher adherence to the high fat pattern in the crude model exhibited greater odds of experiencing headaches compared to those with lower adherence (OR:0.41; 95% CI: 0.17,0.98). However, this significance disappeared after adjusting for confounders in models 1 and 2.

**Table 4:** Associations between nutrient patterns and sign and symptoms of COVID-19 (*n* = 107).

	High carbohydrate, high minerals pattern <−0.04	*p*-value	High protein, high vitamins pattern <−0.17	*p*-value	High fat pattern <0.13	*p*-value	Poor in nutrients pattern <−0.08	*p*-value
OR (95%)								
Variables								
Chest pain (yes)								
Crude	1.78 (0.77,4.15)	0.17	1.78 (0.77,4.15)	0.17	1.59 (0.54,4.65)	0.39	1.64 (0.70,3.80)	0.24
Model 1	1.12 (0.48,2.60)	0.77	1.65 (0.70,3.86)	0.24	0.50 (0.20,1.23)	0.13	1.63 (0.68,3.88)	0.26
Model 2	1.21 (0.50,2.94)	0.66	1.98 (0.80,4.90)	0.13	0.51 (0.20,1.33)	0.17	1.80 (0.71,4.54)	0.21
Headache (yes)								
Crude	1.47 (0.68,4.03)	0.17	1.78 (0.77,4.15)	0.17	0.41 (0.17,0.98)	**0.04**	0.88 (0.01,1.72)	**0.04**
Model 1	1.83 (0.78,4.29)	0.16	1.37 (0.59,3.21)	0.45	0.56 (0.23,1.34)	0.19	1.85 (0.77,4.40)	0.16
Model 2	1.94 (0.77,4.87)	0.15	1.56 (0.63,3.88)	0.33	0.60 (0.23,1.55)	0.59	2.26 (0.88,5.79)	**0.05**
Nose drip (yes)								
Crude	0.81 (0.34,1.96)	0.63	1.18 (0.50,2.78)	0.69	0.43 (0.18,1.04)	0.06	0.72 (0.30,1.70)	0.46
Model 1	1.14 (0.59,3.34)	0.43	0.95 (0.40,2.26)	0.91	0.54 (0.22,1.31)	0.17	0.77 (0.31,1.88)	0.56
Model 2	1.15 (0.45,2.95)	0.75	0.79 (0.30,2.03)	0.62	0.51 (0.19,1.34)	0.17	0.76 (0.29,1.99)	0.57
Vomit (yes)								
Crude	0.77 (0.23,2.54)	0.66	1.10 (0.34,3.60)	0.86	2.63 (0.74,9.30)	0.13	1.68 (0.50,5.62)	0.39
Model 1	1.16 (0.33,3.99)	0.81	1.95 (0.55,6.90)	0.30	1.47 (0.40,5.40)	0.56	1.38 (0.39,4.93)	0.61
Model 2	2.06 (0.79,5.36)	0.13	2.58 (0.66,10.05)	0.17	1.17 (0.44,3.07)	0.74	1.68 (0.42,6.61)	0.45
Nausea (yes)								
Crude	1.06 (0.46,2.47)	0.87	1.28 (0.55,2.98)	0.56	0.86 (0.37,2.00)	0.86	1.67 (0.71,3.90)	0.23
Model 1	1.97 (0.83,4.70)	0.12	1.10 (0.46,2.60)	0.82	1.03 (0.42,2.50)	0.94	1.70 (0.70,4.12)	0.23
Model 2	1.31 (0.33,5.08)	0.69	1.19 (0.46,3.03)	0.71	1.69 (0.42,6.67)	0.45	1.26 (0.42,3.74)	0.66
Diarrhea (yes)								
Crude	1.03 (0.38,2.76)	0.94	1.33 (0.49,3.57)	0.56	0.90 (0.34,2.42)	0.90	1.82 (0.67,4.97)	0.23
Model 1	1.47 (0.52,4.13)	0.45	1.74 (0.65,4.93)	0.29	0.61 (0.21,1.80)	0.38	1.71 (0.59,4.96)	0.31
Model 2	1.22 (0.38,3.93)	0.73	0.25 (0.77,8.39)	0.12	0.64 (0.20,0.04)	0.45	2.44 (0.73,8.16)	0.14
Sore throat (yes)								
Crude	0.58 (0.23,1.47)	0.25	1.13 (0.45,2.82)	0.79	0.66 (0.26,1.67)	0.38	0.77 (0.31,1.94)	0.59
Model 1	1.12 (0.45,2.77)	0.80	0.96 (0.38,2.40)	0.93	0.80 (0.34,2.25)	0.79	1.00 (0.39,2.58)	0.99
Model 2	0.97 (0.36,2.61)	0.95	0.75 (0.24,2.08)	0.58	0.97 (0.35,2.71)	0.96	1.08 (0.38,3.05)	0.88
Joints pain (yes)								
Crude	1.35 (0.56,3.22)	0.49	1.33 (0.55,3.18)	0.51	0.96 (0.40,2.29)	0.93	1.53 (0.61,3.66)	0.33
Model 1	1.07 (0.44,2.60)	0.86	0.79 (0.32,1.93)	0.60	1.37 (0.55,3.44)	0.49	0.93 (0.38,2.30)	0.88
Model 2	1.05 (0.41,2.61)	0.92	0.80 (0.32,2.01)	0.64	1.38 (0.53,3.59)	0.50	0.98 (0.38,2.05)	0.96
Stomach ache (yes)								
Crude	1.93 (0.64,5.86)	0.23	1.42 (0.49,4.16)	0.51	1.59 (0.54,4.65)	0.39	2.04 (0.68,6.13)	0.20
Model 1	1.27 (0.43,3.74)	0.66	1.95 (0.63,5.93)	0.24	0.80 (0.26,2.45)	0.70	1.59 (0.52,4.86)	0.41
Model 2	4.92 (0.43,55.64)	0.19	1.17 (0.68,6.94)	0.18	0.89 (0.27,2.91)	0.85	2.02 (0.59,6.86)	0.26
Confusion (yes)								
Crude	0.90 (0.36,2.26)	0.83	1.13 (0.49,2.61)	0.76	0.83 (0.33,2.07)	0.69	1.50 (0.59,3.77)	0.38
Model 1	1.43 (0.56,3.61)	0.68	1.39 (0.54,3.55)	0.49	0.42 (0.15,1.16)	0.09	0.96 (0.37,2.46)	0.93
Model 2	1.40 (0.53,3.68)	0.49	1.41 (0.53,3.70)	0.48	0.45 (0.16,1.28)	0.13	0.96 (0.37,2.65)	0.99
Contusion								
Crude	0.76 (1.13,2.63)	0.76	1.13 (0.45,2.82)	0.79	0.95 (0.41,2.20)	0.92	1.66 (0.70,3.93)	0.34
Model 1	2.05 (0.87,4.85)	0.10	1.13 (0.48,2.64)	0.77	1.31 (0.54,3.16)	0.53	0.96 (0.37,2.46)	0.93
Model 2	1.93 (0.78,4.76)	0.14	1.02 (0.42,2.47)	0.96	1.41 (0.56,3.59)	0.46	1.78 (0.66,4.83)	0.25
Fever (yes)								
Crude	0.87 (0.34,2.23)	0.77	1.37 (0.53,3.52)	0.50	0.77 (0.30,1.97)	0.58	2.06 (0.79,5.45)	0.13
Model 1	1.31 (0.51,3.39)	0.56	0.67 (1.22,0.47)	0.67	1.46 (0.55,3.89)	0.44	3.09 (1.11,8.56)	**0.03**
Model 2	1.41 (0.50,3.92)	0.50	1.35 (0.48,3.76)	0.56	1.87 (0.62,5.59)	0.26	3.0.09 (1.11,8.58)	**0.03**
Chills (yes)								
Crude	0.84 (0.35,1 97)	0.69	0.84 (0.35,1.97)	0.69	0.87 (0.37,2.05)	0.76	1.66 (0.70,3.93)	0.24
Model 1	1.18 (0.50,2.82)	0.69	0.61 (0.80,0.33)	0.61	1.49 (0.60,3.68)	0.38	1.57 (0.60,3.76)	0.30
Model 2	1.18 (0.46,2.97)	0.72	0.88 (0.35,2.24)	0.79	1.82 (0.67,4.93)	0.23	2.10 (0.73,6.07)	0.16
RDS (yes)								
Crude	0.80 (0.29,2.24)	0.67	1.04 (0.38,2.89)	0.92	0.38 (0.13,1.13)	0.08	0.36 (0.12,1.07)	**0.06**
Model 1	0.98 (0.34,2.79)	0.97	1.32 (0.46,3.81)	0.60	0.20 (0.06,0.71)	**0.01**	0.24 (0.07,0.80)	**0.02**
Model 2	0.72 (0.22,2.33)	0.58	1.26 (0.40,3.88)	0.68	0.18 (0.05,0.65)	**0.01**	0.22 (0.05,0.84)	**0.02**

DH, Duration of hospitalization; RDS, Respiratory distress syndrome; CRP, C-reactive protein; Data were obtained from the binary regression. Model 1 was adjusted for age, sex, education level, BMI and physical activity. Model 2 was adjusted age, sex, education level, BMI, physical activity, comorbidity, use of medication, and supplementation use. Nutrient patterns were reported as median. High carbohydrate, high minerals ≥ −0.08, high protein, high vitamins ≥ 0.013, high fat ≥ −0.017 and Poor in nutrients ≥ −0.04 are considered as a reference category. Data are presented as OR and 95% confidence interval. Model 1 adjusted for sex, marital, status, education, job, disease history, medication use, and supplementation intake. Significant *P* value < 0.05 are bolded. In addition, *P*-values between 0.05 and 0.07 were considered marginally significant. In binary logistic regression chest pain (No), headache (No), nose (No), vomiting (No), nausea (No), diarrhea (No), sore throat (No), stomach pain (No), joint pain (No), confusion (No), contusion (No), chills (NO), RDS (No) considered as a reference group.

Higher odds of headache in model 1 (OR:3.09; 95% CI: 1.11,8.56) and model 2 (OR:3.09; 95% CI: 1.11,8.58), was observed with higher adherence to poor nutrient pattern. A higher adherence to poor nutrients pattern was associated with higher odds of RDS in the crude model (OR:0.36; 95% CI:0.12,1.07). Model 1 (OR:0.24; 95% CI:0.07,0.80), and model 2 (OR:0.22; 95% CI: 0.05,0.84). There were significant higher odds of RDS in individuals with higher fat pattern in model 1 (OR:0.20; 95% CI: 0.06,0.71), and model 2 (OR:0.18; 95% CI: 0.05,0.65).

## 4 Discussion

This study is the first to examine the association between nutrient patterns and COVID-19 severity and hospitalization duration in Iranian adults. Our findings showed that higher adherence to poor dietary patterns is associated with longer hospital stays, reduced appetite, and a higher likelihood of experiencing headaches, fever, and respiratory distress syndrome. Specifically, this study indicated that a high-fat diet was strongly associated with respiratory distress and headaches.

In line with our findings, a recent study in Iran, found that following a healthy dietary pattern before a COVID-19 diagnosis was associated with lower inflammatory markers and a reduced risk of severe COVID-19, hospitalization, and prolonged recovery. In contrast, adherence to unhealthy dietary patterns was related to higher inflammatory markers and an increased risk of severe COVID-19 and extended hospitalization. These diets were strongly associated with symptoms including cough, fever, chills, weakness, muscle aches, nausea, vomiting, and sore throat, while healthy diets were connected to a lower likelihood of shortness of breath, weakness, and sore throat (39). Similarly, adherence to the Mediterranean diet, known for its emphasis on plant-based foods and healthy fats, was correlated with a lower risk of severe COVID-19 symptoms, as well as a lower risk of cardiovascular disease and many other chronic conditions (34).

A cross-sectional study on 250 COVID-19 Iranian patients, higher Mediterranean diet score was associated with reduced symptoms, including dyspnea, cough, fever, chills, weakness, myalgia, nausea and vomiting, sore throat, and shorter hospital stays, faster recovery, and lower inflammatory biomarkers (40). Furthermore, A study involving healthcare workers (HCWs) from six countries (France, Italy, the UK, Spain, Germany, and the USA) with substantial COVID-19 exposure revealed that individuals adhering to plant-based or pescatarian diets exhibited a lower risk of experiencing moderate-to-severe COVID-19. In contrast, those following low-carbohydrate, high-protein diets demonstrated a higher risk. However, no significant association was observed between self-reported dietary patterns and either COVID-19 infection or the duration of illness. It is worth noting that this study had limitations, such as a male-only population, exclusion of severe COVID-19 cases, and variability in the definitions and interpretations of dietary patterns across countries (36). The exact mechanisms by which dietary patterns affect COVID-19 severity and symptoms remain unclear. However, specific micronutrients, such as Vitamins A, B6, C, and D, are vital for supporting the immune system and may influence COVID-19 outcomes (39). Poor nutrition can lead to deficiencies in essential vitamins and minerals, which play a significant role in the severity and progression of diseases like COVID-19. For instance, a deficiency in vitamin D can weaken immune function and increase the risk of contracting COVID-19. Additionally, omega-3 fatty acids found in fatty fish can help reduce systemic inflammation and aid immune response, Vitamin E deficiency has been linked to lipid peroxidation (41, 42). Dietary fiber, abundant in fruits and vegetables, supports gut microbiota and the production of short-chain fatty acids, contributing to lower inflammation. These factors can influence the severity of diseases and the length of hospitalization for COVID-19 patients (43, 44). In COVID-19 patients, cytokine storm incited by viral infections leads to multi-organ failure (45). Furthermore, Gut flora, which ferments dietary fibers and produces anti-inflammatory short-chain fatty acids, plays a role in modulating immune system response (16). Studies uncovered a correlation between poor dietary patterns, prolonged hospitalization, and diminished appetite. A systematic review by Smith et al. showed that high-fat diets promote chronic inflammation, which may contribute to severe COVID-19 outcome (46). Consistent with our finding, poor dietary patterns, particularly those high in fat, are associated with prolonged hospital stays and more severe symptoms of COVID-19.

Our findings highlighted a strong relationship between high-fat dietary patterns and an increased risk of respiratory distress syndrome (ARDS) and headaches. ARDS, driven by excessive immune response and systemic inflammation, may be alleviated by anti-inflammatory diets (47). Prior research indicates that higher consumption of cholesterol or saturated fats triggers acute inflammatory responses and activates inflammatory pathways, such as NOD-like receptors (NLRs) and toll-like receptors (TLRs). Furthermore, saturated fatty acids (SFAs) may elevate ACE2 expression, the receptor facilitating SARS-CoV-2 entry into cells, potentially influencing COVID-19 progression (48–53).

This study is the first to investigate the association between dietary patterns and COVID-19 severity and hospitalization duration in Iranian adults, revealing that poor dietary habits, particularly high-fat diets, are associated with longer hospital stays and more severe symptoms. While the findings are promising, it is essential to acknowledge the limitations, including its cross-sectional design, which limits the ability to establish cause-and-effect relationships. Furthermore, the reliance on self-reported dietary data may introduce reporting bias and inaccurate portion size reporting. Moreover, this study focus on a single hospital and a specific population may limit the generalizability of the findings to other population. Future research with prospective designs, larger and more diverse populations, and objective dietary assessment methods, such as food frequency questionnaires or dietary records, is warranted to confirm these findings and provide a more robust understanding of the relationship between nutrient patterns and COVID-19 outcomes.

Our comprehensive investigation has unveiled a compelling correlation between adherence to an unhealthy dietary pattern, prolonged hospitalization, and decreased appetite. Furthermore, adherence to a poor dietary pattern has been shown to elevate the probability of experiencing symptoms such as headache, fever, and respiratory distress syndrome. Our findings revealed a strong association between ARDS and headache with adherence to a high-fat dietary pattern.

Given the promising findings of this study, future research could further explore the relationship between nutrient patterns and COVID-19 severity in different populations, considering factors such as age, gender, and underlying health conditions. Additionally, longitudinal studies with larger sample sizes, including multi-hospital or international studies, could provide deeper insights into the long-term impact of dietary patterns on COVID-19 outcomes and enhance the generalizability of the findings. Future studies could also incorporate more detailed dietary assessment methods, such as food frequency questionnaires (FFQ), to better capture habitual dietary intake and refine the nutrient patterns associated with disease severity. Furthermore, future research should consider additional confounders, such as income, smoking habits, and long-term dietary patterns, to better understand their potential influence on COVID-19 outcomes. Finally, the inclusion of objective health markers, such as inflammatory levels or oxygen saturation, would help provide a more comprehensive understanding of the relationship between diet and disease severity.

## Data Availability

The datasets presented in this article are not readily available due to restrictions on data sharing. Access to the data is limited to authorized personnel or requires prior approval from the corresponding author or institution. Requests to access the datasets should be directed to mirzaei_kh@sina.tums.ac.ir.

## Glossary

ACE: Angiotensin-Converting Enzyme
ANCOVA: Analysis of covariance
ANOVA: Analysis of variance
ARDS: Acute Respiratory Distress Syndrome
BMI: Body mass index
CI: Confidence interval
COVID-19: Coronavirus disease
CRP: C-reactive protein test
CT scan: Computed tomography scan
ER: Endoplasmic reticulum
HIV: Human Immunodeficiency Virus
IL-6: Interleukin 6
IPAQ: International Physical Activity Questionnaire
KMO: Kaiser-Meyer-Olkin
NLRs: NOD-like receptors
OR: Odds ratio
PA: Physical activity
PCA: Principal component analysis
PKC: Protein Kinase C
ROS: Reactive oxygen species
RT-PCR: Reverse transcription polymerase chain reaction
SARS-CoV-2: Severe Acute Respiratory Syndrome COronaVirus 2
SD: Standard deviation
SFAs: Saturated fatty acids
TLR: Toll-like receptor
WHO: World Health Organization
